# Inequalities in changing mortality and life expectancy in Jiading District, Shanghai, 2002–2018

**DOI:** 10.1186/s12889-021-10323-9

**Published:** 2021-02-05

**Authors:** Qian Peng, Na Zhang, Hongjie Yu, Yueqin Shao, Ying Ji, Yaqing Jin, Peisong Zhong, Yiying Zhang, Yingjian Wang, Shurong Dong, Chunlin Li, Ying Shi, Yingyan Zheng, Feng Jiang, Yue Chen, Qingwu Jiang, Yibiao Zhou

**Affiliations:** 1 Jiading District Center for Disease Control and Prevention, Shanghai, 201800 China; 2 Fudan University School of Public Health, Building 8, 130 Dong’an Road, Xuhui District, Shanghai, 200032 China; 3 Key Laboratory of Public Health Safety, Fudan University, Ministry of Education, Building 8, 130 Dong An Road, Xuhui District, Shanghai, 200032 China; 4 Fudan University Center for Tropical Disease Research, Building 8, 130 Dong’an Road, Xuhui District, Shanghai, 200032 China; 5 School of Epidemiology and Public Health, Faculty of Medicine, University of Ottawa, Ottawa, Canada

**Keywords:** Age-standardized mortality rate, Cause of death, Health inequality, Life expectancy

## Abstract

**Background:**

Improvements of population health in China have been unevenly distributed among different sexes and regions. Mortality Registration System provides an opportunity for timely assessments of mortality trend and inequalities.

**Methods:**

Causes of death were reclassified following the method of Global Burden of Disease Study (GBD). Age-standardized mortality rate (ASMR) and ring-map of the rate by town were used to describe inequalities in changing mortality. Life expectancy (LE) and cause-deleted LE were calculated on the basis of life table technique.

**Results:**

The burden of death from 2002 to 2018 was dominated by cardiovascular diseases (CVD), neoplasms, chronic respiratory diseases and injuries in Jiading district, accounting for almost 80% of total deaths. The overall ASMR dropped from 407.6/100000 to 227.1/100000, and LE increased from 77.86 years to 82.31 years. Women lived about 3.0–3.5 years longer than men. Besides, a cluster of lower LE was found for CVD in the southeast corner and one cluster for neoplasms in the southern corner of the district. The largest individual contributor to increment in LE was neoplasms, ranged from 2.41 to 3.63 years for males, and from 1.60 to 2.36 years for females.

**Conclusions:**

Improvement in health was mainly attributed to the decline of deaths caused by CVD and neoplasms, but was distributed with sex and town. This study served as a reflection of health inequality, is conducive to formulate localized health policies and measures.

**Supplementary Information:**

The online version contains supplementary material available at 10.1186/s12889-021-10323-9.

## Introduction

The world health statistics published annually since 2005 monitor progress in the state of the world’s health and identify inequalities, global life expectancy (LE) at birth for both sexes increased by 5.5 years, ranged from 66.5 years in 2000 to 72.0 years in 2016 [1]. Cause-specific mortality rate and LE have been widely adopted to reflect the health status of residents and reveal mortality trends and disparities all over the world [2–5]. The Global Burden of Diseases, Injuries, and Risk Factors Study (GBD) in 2017 reported the death rate between 2007 and 2017 decreased globally by 7.9% for non-communicable diseases (NCDs), 31.8% for communicable, maternal, neonatal and nutritional diseases (CMNN), and 13.7% for injuries, respectively [6]. Foreman K. J predicted that global LE will increase 4.4 years for both men and women by 2040 [7]. However, the improvement of global health varies substantially among different sexes, ages, and countries. A systematic analysis for the global burden of disease found significant disparities between men and women in terms of age-standardized mortality rate (ASMR) by cause [8]. A study predicted that Japan, Singapore, Spain, and Switzerland would have a projected LE exceeding 85 years while Lesotho, Somalia, and Zimbabwe would have predicted LE below 65 years by 2040 [7].

In mainland China, mortality rate has being reducing and LE has been improving continuously since 1949. Infant mortality, a globally recognized indicator for health and socioeconomic status, has also declined substantially [9]. Previous research found that the annual percentage change in ASMR from 2004 to 2016 was 1.98% of for males and 2.45% for females [10]. However, significant heterogeneity in LE was found among provinces [11]. Shanghai has been ranked first in LE in China for decades with a 6.5 years above the national average in 2016 [12], but there remains inequality among sub-populations and improvement in health has been unevenly distributed. As one of the larger suburbs in Shanghai, the demographic characteristics and social development of Jiading District are obviously different from the overall of Shanghai (Table S1). However, there is a lack of mortality and disease burden studies in the suburbs, let alone studies to assess inequality among towns. Regional studies are significant in discovering health inequality and providing guidance for policy and health resources allocation. Therefore, we conducted this study in Jiading District to examine the inequality in changing mortality and LE between sexes and geographical locations.

## Methods

### Study area

This study was conducted in Jiading District, which is located in the northwest of Shanghai with abundant precipitation, warm and humid climate, moderate light temperature and adequate sunshine. The district covers an area of 464.2 km^2^ and 12 towns with a registered population of 658,200 in 2018. The environment and socioeconomic status are unevenly distributed among different towns in Jiading District. The traffic lines mainly congregate in the southeast (Figure S1), making it more likely exposed to traffic-related pollutants. The central towns of Jiading District are more developed, while the north, northwest and northeast are underdeveloped. Government report in 2015 showed the annual per capital income ranged from 3074.9 to 7035.8 dollars among different towns in Jiading District. Besides, three large hospitals in Jiading District are mainly distributed in the central of this district.

### Data collection

The Full-Scale Mortality Registration System in Shanghai was established in 1973, and the database is maintained and controlled by the Centers for Disease Control and Prevention in Shanghai. The causes of death are currently coded by the International Statistical Classification of Diseases,10th revision (ICD-10). In this study, death data and total population data of Jiading District from 2002 to 2018 were collected. Since the population data by town has been collected by the public security bureau in Jiading district since 2012, we can only conduct death analysis from 2012 to 2018 within towns. We extracted gender, age, death time, underlying cause of death, and address at time of death for each deceased person.

### GBD cause list

Currently, the GBD classification criteria for causes of death is commonly used, the GBD study has divided causes of death into four levels by a hierarchical method [13]. Level 1 contains three broad categories including CMNN, NCDs and injuries. Level 2 subdivides the causes of death at level 1 into 21 categories, such as cardiovascular diseases (CVD), neoplasms, chronic respiratory diseases, neurological disorders, transport injuries. The following levels continue to subdivide cause list in level 2. In this study, causes of death coded by ICD-10 were reclassified in line with the GBD classification criteria. Detailed method was shown in Table S2 which demonstrated the list of ICD codes mapped to the GBD cause list. Garbage codes which cannot be underlying causes of death were classified as other diseases.

### Analytical methods

#### ASMR

The total number of death in each category was analyzed to calculate the proportion of cause of death during study period. The population data in Jiading district was tabulated by age group (0-, 5-, 10-, 15-,20-, …,85-) and by gender, and then the Chinese standard population in 2010 was used as reference to calculate annually cause-special ASMR in Jiading district from 2002 to 2018 and cause-special ASMR by town from 2012 to 2018. The ring-map of ASMR by town was drawn by using ArcMap10.5(Environmental Systems Research Institute,Inc.,Redlands,CA).

#### LE and cause-deleted LE

We utilized the life table technique as fundamental technique to evaluate the impact of cause-special death on human life. LE at birth was calculated by using abridged life table [14]. Then, cause-deleted life table was developed to estimate the impact of cause-special deaths on LE of residents in Jiading District [15], which quantified the number of years added to LE under different scenarios of death elimination.

## Results

### The proportion of cause of death

**Fig. 1:**
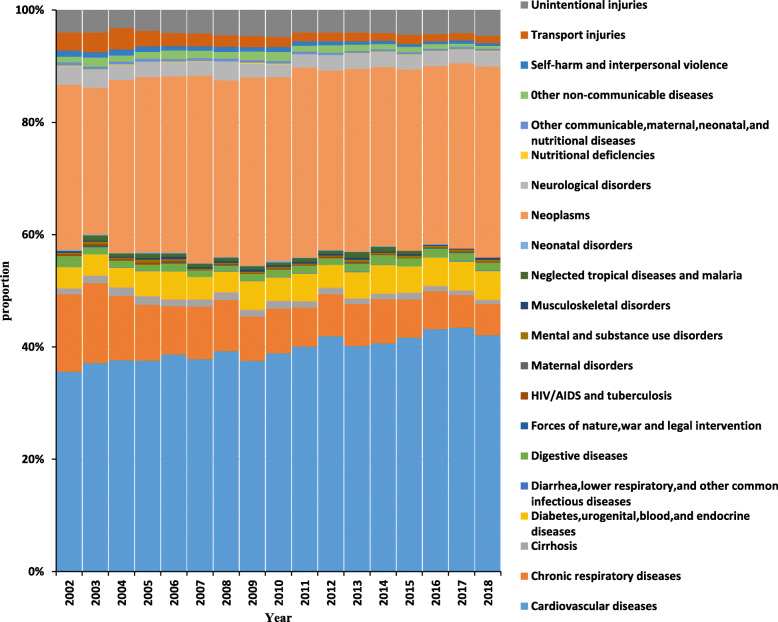
The proportion of the cause of deaths in Jiading district from 2002 to 2018

There were a total of 75,360 deaths in Jiading District from 2002 to 2018, of these, 52.1% were men and 82.3% were the elderly (≥65 years). Except for 6988 deaths due to unknown causes, the proportion of deaths with definite causes at the broad category (Level 2) in Jiading district from 2002 to 2018 was shown in Fig. 1. The top four causes of death in Jiading district were CVD, neoplasms, chronic respiratory diseases and injury. CVD comprised the biggest fraction of deaths (40.0% on average). The proportion of CVD increased from 35.6% in 2002 to 43.6% in 2017, and slightly leveled off in 2018 (42.3%). Neoplasms as cause of death varied in proportions between 29.4 and 33.6% in 2009. The proportion of death from chronic respiratory diseases declined from 13.8 to 5.6%. All injury ranked 4st among the all-cause deaths, and the proportion had been in decline during this period. Details of cause-special number of deaths and proportion were described in Table S3.

### ASMR

**Fig. 2:**
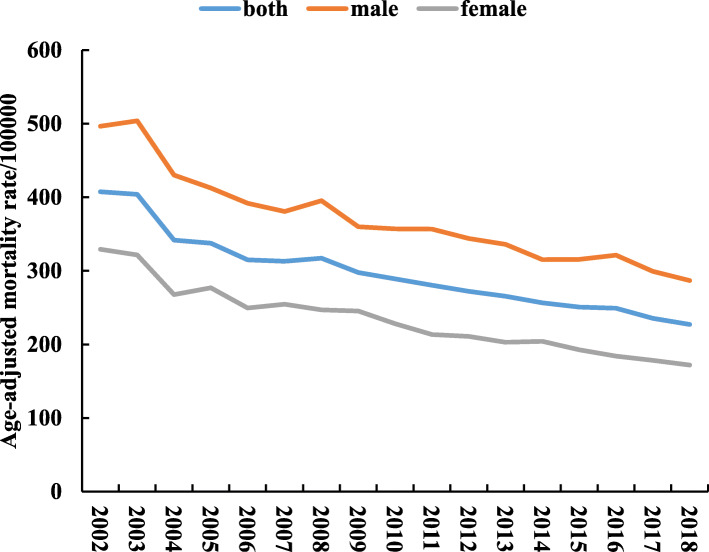
Age-adjusted mortality rate from 2002 to 2018 in Jiading district

The results of ASMR in Jiading district from 2002 to 2018 were presented in Fig. 2. The overall ASMR dropped from 407.6/100000 in 2002 to 227.1/100000 in 2018. The most significant drop appeared between 2003 and 2004 with a gap of 62.0/100000. In addition, the male ASMR was generally higher than that of female.

**Fig. 3:**
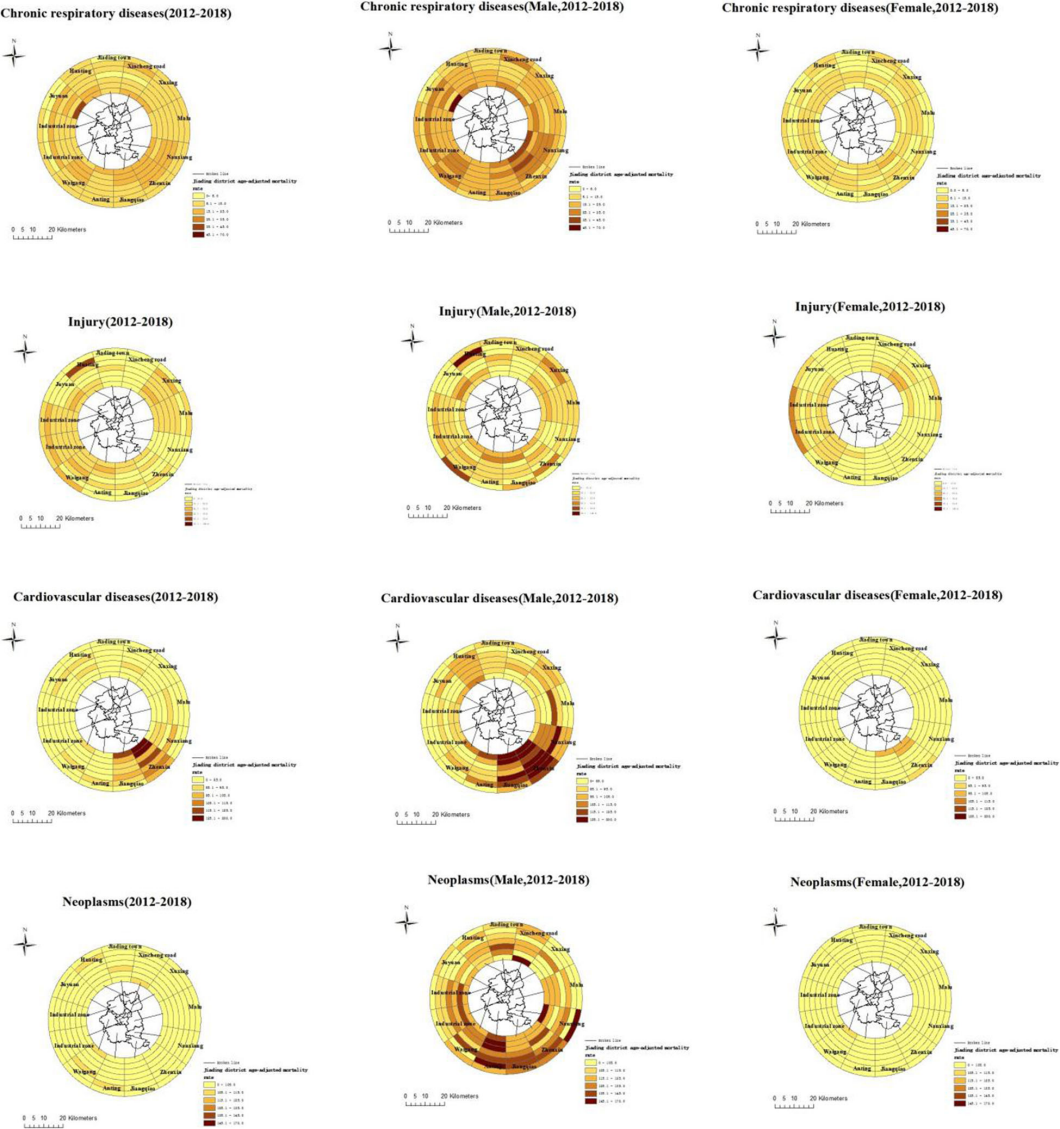
The ring map of age-standardized mortality rates for chronic respiratory diseases, injury, cardiovascular diseases and neoplasms by town and gender in Jiading district from 2012 to 2018

Figure 3 displays the ring map of ASMR for chronic respiratory diseases, injury, CVD and neoplasms by town in Jiading district from 2012 to 2018. ASMR in males were generally higher and more volatile than that in females, and there were different death clusters or trends according to the top four causes of death.

For CVD, the rate was clustered in the southeast corner of Jiading district (towns of Jiangqiao, Zhenxin and Nanxiang), particularly for males, and decreased most for Zhenxin town from 197.1/100000 in 2012 to 117.9/100000 in 2018. For neoplasms, the mortality was clustered in the southern corner of Jiading district (towns of Anting, Jiangqiao and Zhenxin), but in males only.

The ASMR for chronic respiratory diseases were generally less than 20/100,000 except for certain years in Juyuan zone, Waigang town and Zhenxin town and decreased most in males of Nanxiang town and Zhenxin town. The ASMR for injury was less than 45.0 per 100,000 most years, but was markedly higher for the towns of Huating town (105.3/100000) in 2017 and Waigang (107.5/100000) in 2018.

### LE

**Fig. 4:**
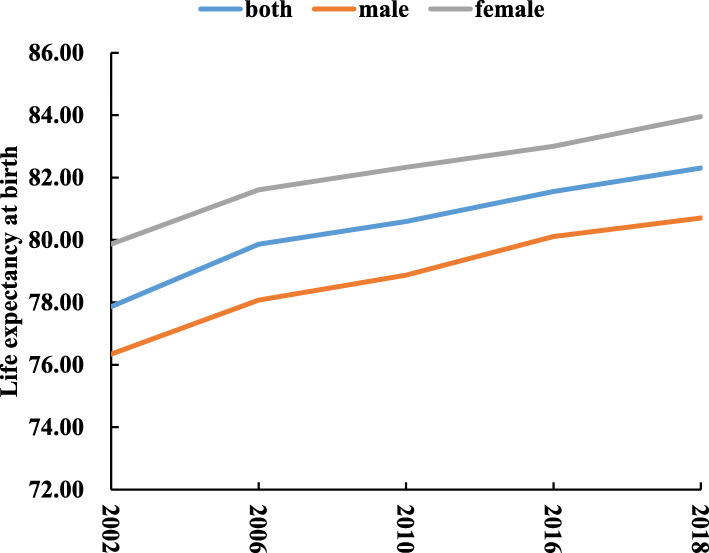
Life expectancy at birth in Jiading district from 2002 to 2018

Figure 4 showed the trend of LE in Jiading district from 2002 to 2018, LE at birth ranged from 77.86 years in 2002 up to 82.31 years in 2018, and the most obvious increase appeared between 2002 and 2006, with a gap of 2 years. Meanwhile, LE was generally longer in females than males with a difference 2.90–3.53 years for the study years.

**Fig. 5:**
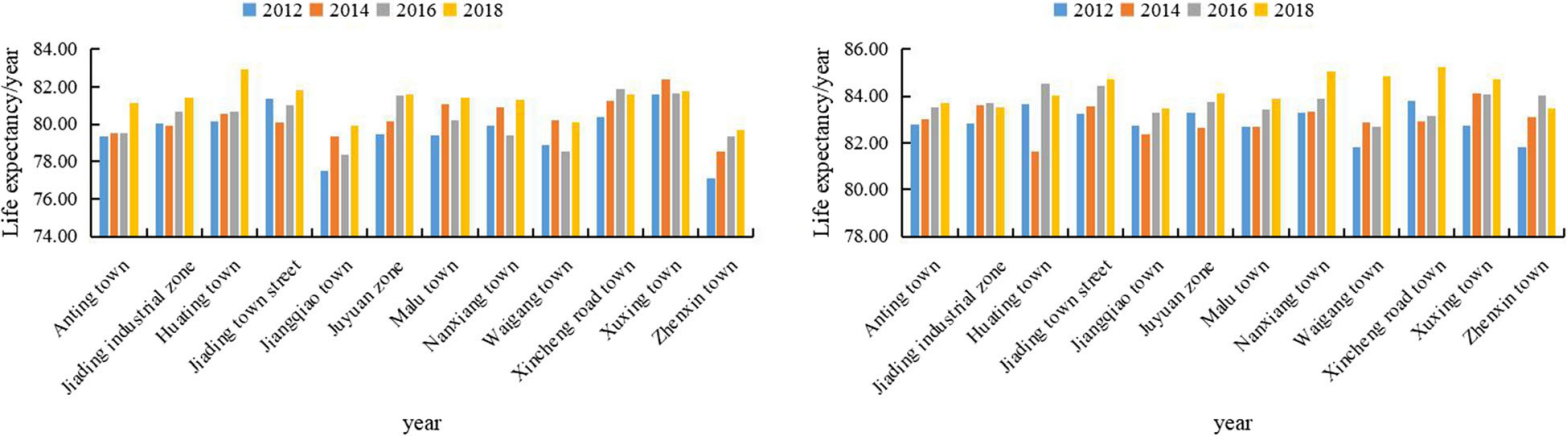
Change of life expectancy in Jiading district from 2012 to 2018 for males (left) and female (right)

Figure 5 showed the LE from 2012 to 2018 by town. For males, LE ranged from 77.12 years in Zhenxin to 81.57 years in Xuxing in 2012, and from 79.71 years in Zhenxin to 82.91 years in Huating in 2018. For females, LE varied from 81.80 years in Waigang to 83.80 years in Xincheng Road in 2012, and from 83.46 years in Jiangqiao to 85.23 years in Xincheng Road in 2018.

### Cause-deleted LE

Table 1 provided an assessment of increments in LE by town if chronic respiratory disease, injury, CVD and neoplasm were completely eradicated in males, respectively. Overall, the largest individual contributor to increment in LE was neoplasm, followed by cardiovascular disease, injury and chronic respiratory disease. Total increments in LE due to the four causes were higher for the towns of Zhenxin, Jiangqiao and Nanxiang (8.0 years on average), and were the lowest for Xuxing, with an average of 6.18 years. We noted there was a tendency for gradual decline or fluctuate decrease of total increments in LE from 2012 to 2018. The average increments in LE caused by neoplasm ranged from 2.41 years in Xuxing to 3.63 years in Anting. The increments in LE due to CVD were higher in Zhenxin, Jiangqiao and Nanxiang, with average gains of 3.07, 2.74, 2.44 years, respectively.

**Table 1:** Loss of life expectancy due to Chronic Respiratory Disease, Injury, Cardiovascular Disease and Neoplasm by Town in Males

	Chronic respiratory diseases				Injury				Cardiovascular diseases				Neoplasms				Total			
	2012	2014	2016	2018	2012	2014	2016	2018	2012	2014	2016	2018	2012	2014	2016	2018	2012	2014	2016	2018
Anting town	0.37	0.39	0.40	0.49	0.51	0.56	0.53	0.34	2.56	1.84	1.9	1.94	3.39	4.05	3.68	3.42	8.41	8.12	7.84	6.97
Jiading industrial zone	0.44	0.43	0.75	0.38	0.58	0.52	0.56	0.34	2.05	1.78	1.47	1.82	3.26	3.54	3.09	2.78	7.63	7.43	6.90	6.17
Huating town	0.50	0.24	0.43	0.21	0.51	0.29	0.85	0.53	2.48	2.29	2.44	2.14	2.87	3.19	2.5	1.78	7.74	7.13	7.46	5.42
Jiading town street	0.36	0.13	0.29	0.24	0.25	0.50	0.31	0.39	2.09	2.25	2.31	2.1	2.61	4.1	2.96	2.97	6.18	7.93	6.84	6.06
Jiangqiao town	0.60	0.28	0.30	0.45	0.71	0.24	0.36	0.66	3.26	2.57	2.91	2.22	2.68	3.13	3.33	3.47	8.89	7.28	8.26	8.14
Juyuan zone	1.25	0.56	0.70	0.11	0.42	0.34	0.02	0.18	2.18	1.56	2.55	2.14	2.74	3.27	1.98	3.12	8.35	7.33	6.05	6.33
Malu town	0.48	0.28	0.47	0.27	0.62	0.51	0.59	0.58	2.1	1.83	2.02	1.83	3.3	3.21	3.05	3.1	7.71	6.83	7.28	6.61
Nanxiang town	0.81	0.69	0.49	0.28	0.49	0.45	0.36	1.09	1.87	2.47	3.02	2.4	4.04	2.63	3.42	2.66	8.67	7.70	8.87	6.81
Waigang town	0.44	0.48	0.34	0.53	0.59	0.28	0.48	1.45	2.39	1.59	1.99	1.53	2.92	3.07	4.57	3.26	7.70	6.73	8.77	7.88
Xincheng road town	0.44	0.28	0.33	0.45	0.10	0.41	0.54	0.13	1.61	2.69	1.68	2.34	4.24	3.23	2.83	3.44	7.73	7.84	6.10	7.47
Xuxing town	0.56	0.43	0.38	0.22	1.15	0.59	0.75	0.36	1.94	1.79	2.11	2.17	2.49	2.51	2.21	2.41	6.35	5.97	6.19	6.20
Zhenxin town	0.67	0.40	0.73	0.40	0.67	0.70	0.02	0.21	4.13	2.38	2.95	2.83	2.51	3.15	3.22	3.33	10.22	8.21	8.36	7.92
Total	0.54	0.38	0.43	0.33	0.52	0.48	0.44	0.49	2.52	2.11	2.29	2.12	3.07	3.37	3.19	3.21	8.08	7.52	7.60	7.27

Table 2 illustrated increments in LE by town if chronic respiratory disease, injury, CVD and neoplasm were completely eradicated in females, respectively. Clustering for four diseases was less marketed in females. The towns of Jiangqiao and Waigang showed the biggest increase of LE when removing the four causes of death, with average increment in LE of more than 5 years. The largest contributor to total increment in LE was neoplasms for women, with average increment in LE of more than 2 years. However, the biggest contributor to increment in LE was CVD while the least contributor was neoplasms in Zhenxin town. Besides, the increments in LE due to chronic respiratory disease were generally less than 0.3 years. The majority of increments in LE due to injury were less than 0.5 years.

**Table 2:** Loss of life expectancy due to Chronic Respiratory Disease, Injury, Cardiovascular Disease and Neoplasm by Town in Females

	Chronic respiratory diseases				Injury				Cardiovascular diseases				Neoplasms				Total			
	2012	2014	2016	2018	2012	2014	2016	2018	2012	2014	2016	2018	2012	2014	2016	2018	2012	2014	2016	2018
Anting town	0.30	0.17	0.19	0.07	0.39	0.29	0.38	0.27	1.37	1.90	1.71	1.46	2.22	1.75	1.78	2.66	4.79	4.46	4.49	4.89
Jiading industrial zone	0.10	0.29	0.13	0.25	0.51	0.30	0.41	1.46	2.04	1.54	2.06	1.45	2.23	2.03	1.72	1.28	5.41	4.54	4.77	4.91
Huating town	0.21	0.35	0.08	0.17	0.28	0.25	0.20	0.76	1.64	2.00	2.23	2.09	1.83	2.04	1.41	1.62	4.37	5.03	4.31	4.36
Jiading town street	0.14	0.01	0.04	0.04	0.47	0.17	0.11	0.15	1.66	1.63	1.48	1.77	2.48	2.32	2.01	1.56	5.16	4.50	3.94	3.78
Jiangqiao town	0.15	0.12	0.21	0.15	0.15	0.18	0.35	0.12	2.63	1.92	1.79	1.93	2.09	2.77	2.22	2.15	5.69	5.48	5.12	4.75
Juyuan zone	0.24	0.04	0.15	0.00	0.49	0.37	0.60	0.63	1.92	1.82	1.95	0.79	1.89	2.13	1.90	2.35	5.16	4.82	5.16	4.01
Malu town	0.25	0.25	0.25	0.09	0.36	0.28	0.24	0.76	1.89	1.71	1.87	1.47	2.02	2.19	1.43	1.56	5.06	5.06	4.29	4.29
Nanxiang town	0.07	0.15	0.12	0.04	0.18	0.21	0.26	0.17	2.01	2.31	2.07	1.59	1.89	1.77	1.95	1.59	4.63	4.89	4.90	3.70
Waigang town	0.05	0.28	0.15	0.17	0.40	0.56	0.47	0.26	1.61	1.55	1.86	1.45	3.15	1.83	2.56	1.88	5.85	4.80	5.76	4.17
Xincheng road town	0.29	0.00	0.13	0.23	0.63	0.18	0.20	0.08	1.64	1.75	1.44	0.92	1.75	2.29	2.16	2.17	4.69	4.75	4.22	3.71
Xuxing town	0.22	0.19	0.24	0.11	1.06	0.37	0.23	0.35	1.73	1.58	1.80	2.06	1.75	1.54	1.55	1.60	5.27	4.15	4.35	4.62
Zhenxin town	0.22	0.22	0.30	0.25	0.09	0.20	0.22	0.14	2.69	2.10	2.09	2.47	1.96	1.70	1.42	1.30	5.69	4.72	4.51	4.67
Total	0.18	0.18	0.17	0.13	0.39	0.27	0.33	0.42	1.92	1.83	1.90	1.68	2.17	2.04	1.84	1.84	5.21	4.80	4.72	4.48

## Discussion

### General trends

The results in this study showed that Jiading district experienced significant improvement in population health from 2002 to 2018 in terms of ASMR and LE. The burden of death was dominated by CVD, neoplasms, chronic respiratory diseases and injuries, which accounted for almost 80% of total deaths. The mortality rate showed a rapid decline for both sexes before 2004 and a moderate decline from 2004 to 2018. LE at birth in Jiading district increased from 77.86 years in 2002 to 82.31 years in 2018, which was lower compared with the average level in Shanghai [12].

### Gender and geographical inequality in health

There was obvious gender discrepancy and geographical inequality in mortality. In our study, women were estimated to live about 3.0–3.5 years longer than men. Statistics showed that gender gap of life expectancy (GGLE) was 3.0 years for China while 4.1 years for Shanghai in 2018. Previous study conducted in Shanghai found NCDs played an important role in the narrowing of GGLE [16]. The reduced GGLE in Jiading District can be partially explained by the high proportion of NCDs among men in this district. Besides, previous study highlighted injuries due to traffic accidents and falling in males were the main causes of GGLE [17]. Sparse traffic lines and low traffic volume in Jiading District led to less deaths due to traffic accidents in male and finally reduced GGLE.

Men are more likely to die of any given health deficits, which can be attributed to biology as well as behavior. For example, women are less likely to be attacked by CVD, which can be explained by the protection of estrogen to decrease the levels of low density lipoproteins [18]. Women have a lower risk to suffer from diseases associated with the X-chromosome, since X-related defects are more likely to encoded in recessive genes [19]. GGLE is also related to engagement in risky behaviors (smoking, alcohol drinking, drug addition, hazardous driving) and dietary habits (consumption of fruits and vegetables, low-fat and less salt food) [20, 21]. In addition, a study found that women expended more on health than men do within households, and that preference and investment for health could account for almost 70% of the gender gap [22].

ASMR and LE showed cause-special discrepancy and inequality among 12 towns. For example, the mortality caused by CVD was mainly concentrated in the southeast corner of Jiading district, but the mortality due to neoplasms in southern corner of Jiading district accounted for a relatively higher proportion. Previous studies found traffic-related pollutants can increase the risk of death from CVD [23]. According to the distribution map of traffic lines in jiading district (Figure S1), the traffic lines mainly congregate in the southeast, making it more likely exposed to traffic-related pollutants. Furthermore, LE of males in 2012 showed a difference of 4.45 years between Zhenxin town and Xuxing town. Several studies found social-economic was reversely associated with neonatal mortality [24] and cause-special mortality such as stroke [25]. statistics in Jiading district show that Jiangqiao town and Zhenxin town have lower economic level and higher unemployment rate compared with others, we speculate that the social-economic might play an important part in this geographic aggregation. The impact of social economic status was also observed in other countries. A study conducted in Africa showed that poverty was greatly related to health discrepancy [26]. A study from Italy showed that less educated residents had higher mortality and lower LE [27]. However, Alicandro G found that lung cancer and ischemic heart diseases contributed a higher proportion to the socio-economic inequality in males [28]. In our study area, neoplasms and CVD in the towns of Jiangqiao and Zhenxin should be the focus for decreasing geographical inequality .

### CVD

CVD were estimated to be the major cause of death in Jiading district, accounting for 40.0% of total death on average. The outcomes obtained from our study showed that the loss of LE due to CVD were generally higher in Zhenxin town, Jiangqiao town and Nanxiang town, which resembled with the aggregation area of the CVD. Shanghai entered into the ageing society in 1980s [29]. Since the mortality due to CVD increases with age [30], and aging phenomenon in Jiangqiao, Zhenxin, and Nanxiang town might have an effect on the geographical inequality of death. With a rapid development in Jiading district, the prevalence of hypertension and obesity has also been increasing [31, 32] Hypertension and obesity are vital risk factors for CVD [33, 34], which might explain the increased burden of death due to CVD in Jiading district.

### Neoplasms and other causes of death

Neoplasms appeared to be the most significant cause of loss in LE. A U.S. study showed that cancer caused less deaths but higher share of loss in LE compared with heart disease [35]. Previous studies found neoplasms occurred earlier in the life course than other NCDs [36], the onset age of tumor patients was generally earlier than that of CVD, which caused lasting and serious harm to health of patients and led to more loss of LE. Our results suggest the reduction of neoplasms can significantly improve LE and residents’ health. Overall, the proportion of death due to chronic respiratory diseases in Jiading district was slow descent from 13.8% in 2002 to 5.6% in 2018, similar to the global trend [37]. Low socio-demographic index, smoking, high body mass index and pollution from particulate matter were the major contributors to deaths from chronic respiratory diseases [38, 39]. Jiading district has made great efforts to improve the ecological environment, and statistics showed that average concentration of PM2.5 dropped by 13.0%, and green space increased by 2.99% in 2018 [12], which may play an important part in the decline of chronic respiratory diseases.

### Limitation

Our study had several limitations. First, given the population included in this study were local residents in Shanghai, the data were not sufficient enough to assess potential influence of migration. Second, we did not redistribute garbage codes proportionally by geographical features or demographic characteristics such as sex and age, which might affect the accuracy of outcome measures. Besides, this study was also limited by the amount of data on deaths, especially at the town level. Therefore, we can only conducted this study at the broad category (Level 2) of GBD classification criteria for causes of death rather than subdivided the causes of death further. Third, because of the absence of influencing factors, this study could only allow us to generate some hypotheses for heterogeneity observed.

## Conclusion

In conclusion, mortality decreased and LE increased in Jiading district from 2002 to 2018, but the changes showed sex and geographic differences. We observed a cluster of CVD mortality in the southeast corner of Jiading district and one cluster of neoplasm mortality in the southern corner of the district. To improve population health, it is important to reduce the mortality associated with CVD and neoplasms. The study provides comprehensive updates of mortality trend and health inequality that can be used to guide future researches and allocate investment in health-care resources.

## Supplementary Information

**Additional file 1: Table S1.** Comparison of environment, economic, demographics, education and medical level between Jiading District and Shanghai.

**Additional file 2: Figure S1.** Map of traffic lines in Jiading District.

**Additional file 3: Table S2**. List of International Classification of Diseases (ICD) codes mapped to the Global Burden of Disease cause list for causes of death.

**Additional file 4: Table S3.**

## Data Availability

The data that support the findings of this study are available from Jiading District Center for Disease Control and Prevention. but restrictions apply to the availability of these data, which were used under license for the current study, and so are not publicly available. Data are however available from the authors upon reasonable request and with permission of Jiading District Center for Disease Control and Prevention.

## Abbreviations

LE: Life expectancy
GBD: The Global Burden of Diseases, Injuries, and Risk Factors Study
NCDs: Non-communicable diseases
CMNN: Communicable, maternal, neonatal and nutritional
ICD-10: The International Statistical Classification of Diseases, 10th revision
CVD: Cardiovascular diseases
GGLE: Gender gap of life expectancy
