# Supplementary material for: Inequalities in changing mortality and life expectancy in Jiading District, Shanghai, 2002–2018
Source: BMC Public Health. 2021 Feb 5;21:303. doi: 10.1186/s12889-021-10323-9 (PMC7866752; doi:10.1186/s12889-021-10323-9)
Supplement: Supplementary file 1 — Additional file 1: Table S1. Comparison of environment, economic, demographics, education and medical level between Jiading District and Shanghai. [file 12889_2021_10323_MOESM1_ESM.docx]

Table S1 Comparison of environment, economic, demographics, education and medical level between Jiading District and Shanghai

|  | 2010 | |  | 2018 | |
| --- | --- | --- | --- | --- | --- |
|  | Jiading District | Shanghai |  | Jiading District | Shanghai |
| Environment |  |  |  |  |  |
| Average temperature(℃) | 17.0 | 17.8 |  | 17.6 | 17.7 |
| Extreme high temperature(℃) | 39.3 | 38.6 |  | 37.7 | 37.4 |
| Extreme low temperature(℃) | -5.6 | -6.0 |  | -6.4 | -7.0 |
| Precipitation days (≥1mm) | 131 | 131 |  | 134 | 134 |
| Sunshine duration(h) | 1760.7 | 1306.5 |  | 1896.7 | 1407.9 |
| Distinguished achiever ratio of air quality(%) | 92.1 | 92.1 |  | 78.1 | 81.1 |
| Average PM2.5 concentration(μg/m3) | 86 | 79 |  | 40 | 36 |
| Green area per capita(m2) | 16.2 | 13.0 |  | 18.5 | 8.2 |
| Economic |  |  |  |  |  |
| GDP per capita (yuan) | 54829 | 77275 |  | 119628 | 134982 |
| Disposable income per capita (yuan) | 26611 | 31838 |  | 53545 | 64183 |
| Proportion of primary Industry(%) | 0.5 | 0.7 |  | 0.1 | 0.3 |
| Proportion of secondary Industry(%) | 65.0 | 42.3 |  | 68.1 | 29.8 |
| Proportion of tertiary Industry(%0 | 34.5 | 57.0 |  | 38.1 | 69.9 |
| Demographics |  |  |  |  |  |
| Registered population | 557452 | 1412.32 |  | 640739 | 1447.57 |
| Population density/km2 | 1201 | 14760 |  | 1383 | 15309 |
| Male-female ratio | 0.989 | 0.992 |  | 1.007 | 0.981 |
| Urban population ratio(%) | 83.6 | 89.3 |  | 83.2 | 87.7 |
| Percentage of population over 65 years（%） | 7.2 | 16.0 |  | 22.6 | 24.6 |
| birth rate(‰) | 6.7 | 7.1 |  | 6.9 | 7.2 |
| Natural growth rate(‰) | -1.2 | -2.0 |  | -0.9 | -1.8 |
| Urban unemployment rate(%) | 6.2 | 4.2 |  | 4.3 | 3.6 |
| Education |  |  |  |  |  |
| Higher education rate(%) | 99.5 | 99.9 |  | 99.6 | 99.4 |
| Proportion of college students(%) | 13.7 | 28.5 |  | 19.5 | 27.1 |
| Proportion of secondary school students(%) | 70.4 | 32.8 |  | 67.5 | 31 |
| Proportion of elementary school students(%) | 15.9 | 38.7 |  | 13.0 | 41.9 |
| Medical level |  |  |  |  |  |
| Number of health institution | 145 | 3270 |  | 367 | 5298 |
| Number of hospital staff | 4534 | 135400 |  | 8812 | 206500 |
| Number of doctors | 1778 | 51300 |  | 3493 | 74900 |
| Number of doctors per 10,000 population | 12.1 | 22.0 |  | 21.9 | 31.0 |

Extreme high temperature represented the highest temperature of the year.

Extreme low temperature represented the lowest temperature of the year.

The primary industry mainly represented industries that produce food materials and biological materials, including farming, forestry, animal husbandry, aquaculture and other industries that directly produce natural materials.

The secondary industry mainly represented the processing and manufacturing industries or the manual manufacturing operation, which processes fundamental materials.

Tertiary industry represented other industries other than primary and secondary industries including transportation, communications, commerce, catering, finance, education, public services and other non-material production sectors.
