# Supplementary figures and images for: Inequalities in changing mortality and life expectancy in Jiading District, Shanghai, 2002–2018
Source: BMC Public Health. 2021 Feb 5;21:303. doi: 10.1186/s12889-021-10323-9 (PMC7866752; doi:10.1186/s12889-021-10323-9)

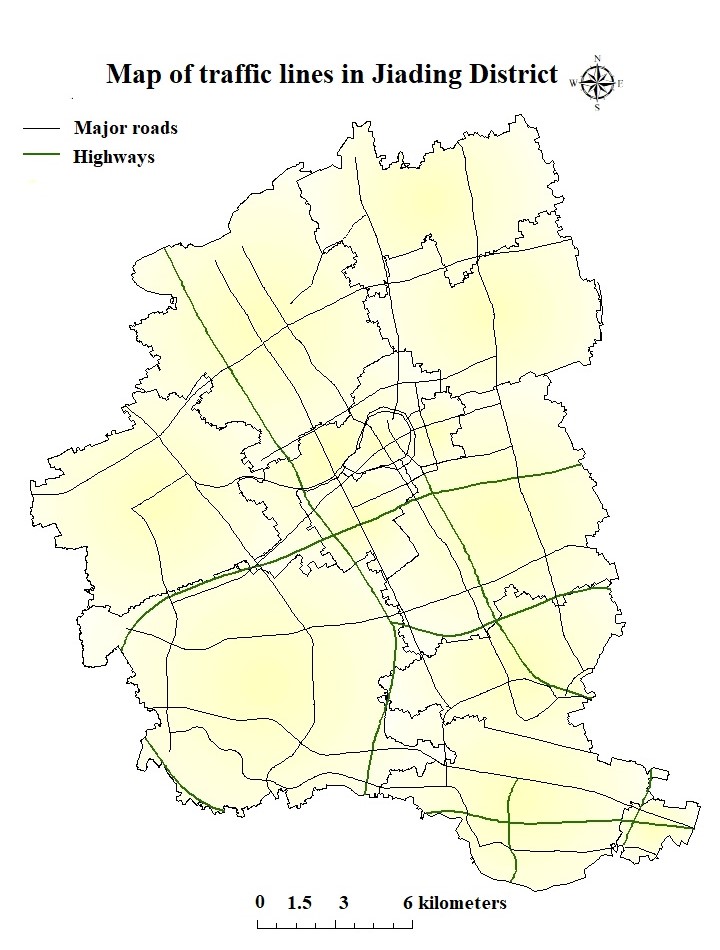

Supplement: Supplementary file 2 — Additional file 2: Figure S1. Map of traffic lines in Jiading District. [file 12889_2021_10323_MOESM2_ESM.jpg]
