# Supplementary material for: Inequalities in changing mortality and life expectancy in Jiading District, Shanghai, 2002–2018
Source: BMC Public Health. 2021 Feb 5;21:303. doi: 10.1186/s12889-021-10323-9 (PMC7866752; doi:10.1186/s12889-021-10323-9)
Supplement: Supplementary file 3 — Additional file 3: Table S2. List of International Classification of Diseases (ICD) codes mapped to the Global Burden of Disease cause list for causes of death. [file 12889_2021_10323_MOESM3_ESM.docx]

| **Table S2. List of International Classification of Diseases (ICD) codes mapped to the Global Burden of Disease cause list for causes of death** | |
| --- | --- |
| **Cause** | **ICD10 Code** |
| Communicable, maternal, neonatal, and nutritional diseases | A00‐A00.9,A01.0‐A14,A15‐A28.9,A31,A31.0,A31.8‐A39.9,A42‐A44.9,A48.1,A48.2,A48.4‐A48.52,A49.1,A50‐A58,A63 A63.8,A65‐A70,A74,A74.8‐A75.9,A77‐A96.9,A98‐A98.8,B00‐B06.9,B10‐B10.89,B15‐B17.2,B19.1‐B19.21,B20‐ B27.99,B29.4,B33‐B33.8,B37‐B37.2,B37.5‐B37.89,B38‐B54.0,B55.0,B56‐B60.8,B63,B65‐B65.9,B67‐B72.0,B74.3‐ B75,B77‐B78.9,B80‐B81.8,B83‐B83.8,B90‐B91,B94.1,B94.2,B95‐B95.5,D50‐D52.0,D52.8‐D53.9,D62‐D63.8,D64.1‐ D64.3,D64.8‐D64.9,D69.9,E00‐E02,E40‐E46.9,E51‐E61.9,E63‐E64.0,E64.2‐E64.9,F07.1,G00.0‐G00.8,G03‐G03.8,G04‐ G05.8,G14,G14.6,H70‐H70.93,I00,I02,I02.9,J01‐J01.91,J02.0,J03.0‐J03.01,J05‐J05.11,J09‐J15.8,J16‐J16.9,J20‐ J21.9,J36,J36.0,K67.0‐K67.8,K74.7,K74.8,K93.0,M03.1,M12.1‐M12.19,M49.0,M49.1,M73.0,M73.1,M89.6‐ M89.69,N70‐N71.9,N73‐N74.8,N96,N98‐N98.9,O00‐O07.9,O09‐O16.9,O20‐O26.93,O28‐O36.93,O40‐O48.1,O60‐ O77.9,O80‐O92.79,O96‐P03.1,P03.6‐P05.9,P07‐P15.9,P19‐P29.9,P35‐P39.9,P50‐P61.9,P70,P70.3‐P72.9,P74‐ P78.9,P80‐P81.9,P83‐P84,P90‐P94.9,P96,P96.3,P96.4,P96.8‐P96.89,R19.7 |
| HIV/AIDS and tuberculosis | A10‐A14,A15‐A19.9,B20‐B24.9,B45‐B45.9,B90‐B90.9,K67.3,K93.0,M49.0,P37.0 |
| Tuberculosis | A10‐A14,A15‐A19.9,B90‐B90.9,K67.3,K93.0,M49.0,P37.0 |
| HIV/AIDS | B20‐B24.9,B45‐B45.9 |
| HIV/AIDS resulting in mycobacterial infection | B20.0 |
| HIV/AIDS resulting in other diseases | B20.1‐B23.9,B24.0,B45‐B45.9 |
| Diarrhea, lower respiratory, and other common infectious diseases | A00‐A00.9,A01.0‐A09.9,A33‐A37.91,A39‐A39.9,A83‐A87.9,B01‐B02.9,B05‐B05.9,B94.1,F07.1,G00.0‐G00.8,G03‐ G03.8,G04‐G05.8,H70‐H70.93,J01‐J01.91,J05‐J05.11,J09‐J15.8,J16‐J16.9,J20‐J21.9,J36,J36.0,P23‐P23.9,P35.8,R19.7 |
| Diarrheal diseases | A00‐A00.9,A02‐A04.1,A04.3,A04.5‐A07,A07.2‐A07.4,A08‐A09.9,R19.7 |
| Cholera | A00‐A00.9 |
| Other salmonella infections | A02‐A02.9 |
| Shigellosis | A03‐A03.9 |
| Enteropathogenic E coli infection | A04.0 |
| Enterotoxigenic E coli infection | A04.1 |
| Campylobacter enteritis | A04.5 |
| Amoebiasis | A06‐A06.9 |
| Cryptosporidiosis | A07.2 |
| Rotaviral enteritis | A08.0 |
| Aeromonas |  |
| Clostridium difficile | A04.7 |
| Norovirus | A08.1‐A08.19 |
| Adenovirus | A08.2 |
| Other bacterial foodborne diarrhea | A05‐A05.9 |
| Other diarrheal diseases | A04,A04.3,A04.6,A04.8,A04.9,A07,A07.3,A07.4,A08,A08.3‐A09.9,R19.7 |
| Intestinal infectious diseases | A01.0‐A01.4,A04.2,A04.4,A07.0,A07.1,A07.8,A07.9 |
| Typhoid fever | A01.0‐A01.09 |

| **Table S2. List of International Classification of Diseases (ICD) codes mapped to the Global Burden of Disease cause list for causes of death** | |
| --- | --- |
| **Cause** | **ICD10 Code** |
| Paratyphoid fever | A01.1‐A01.4 |
| Other intestinal infectious diseases | A04.2,A04.4,A07.0,A07.1,A07.8,A07.9 |
| Lower respiratory infections | J09‐J15.8,J16‐J16.9,J20‐J21.9,P23‐P23.9 |
| Influenza | J09‐J11.89 |
| Pneumococcal pneumonia | J13,J13.0,J15.3,J15.4,J15.6 |
| H influenzae type B pneumonia | J14,J14.0 |
| Respiratory syncytial virus pneumonia | J12.1 |
| Other lower respiratory infections | J12,J12.0,J12.2‐J12.9,J15‐J15.29,J15.5,J15.7,J15.8,J16‐J16.9,J20‐J21.9,P23‐P23.9 |
| Upper respiratory infections | J01‐J01.91,J05‐J05.11,J36,J36.0 |
| Otitis media | H70‐H70.93 |
| Meningitis | A39‐A39.9,A87‐A87.9,G00.0‐G00.8,G03‐G03.8 |
| Pneumococcal meningitis | G00.1 |
| H influenzae type B meningitis | G00.0 |
| Meningococcal meningitis | A39‐A39.9 |
| Other meningitis | A87‐A87.9,G00.2‐G00.8,G03‐G03.8 |
| Encephalitis | A83‐A86.4,B94.1,F07.1,G04‐G05.8 |
| Diphtheria | A36‐A36.9 |
| Whooping cough | A37‐A37.91 |
| Tetanus | A33‐A35.0 |
| Measles | B05‐B05.9 |
| Varicella and herpes zoster | B01‐B02.9,P35.8 |
| Neglected tropical diseases and malaria | A68‐A68.9,A69.2‐A69.5,A75‐A75.9,A77‐A79.9,A82‐A82.9,A90‐A96.9,A98‐A98.8,B50‐B54.0,B55.0,B56‐B58,B59‐B60.8,B65‐B65.9,B67‐B72.0,B74.3‐B75,B77‐B78.9,B80‐B81.8,B83‐B83.8,P37.1,P37.3,P37.4 |
| Malaria | B50‐B54.0,P37.3,P37.4 |
| Chagas disease | B57‐B57.5 |
| Leishmaniasis | B55.0 |
| Visceral leishmaniasis | B55.0 |
| African trypanosomiasis | B56‐B56.9 |
| Schistosomiasis | B65‐B65.9 |
| Cysticercosis | B69‐B69.9 |
| Cystic echinococcosis | B67‐B67.99 |
| Dengue | A90‐A91.9 |
| Yellow fever | A95‐A95.9 |
| Rabies | A82‐A82.9 |
| Intestinal nematode infections | B77‐B77.9 |
| Ascariasis | B77‐B77.9 |

| **Table S2. List of International Classification of Diseases (ICD) codes mapped to the Global Burden of Disease cause list for causes of death** | |
| --- | --- |
| **Cause** | **ICD10 Code** |
| Other neglected tropical diseases | A68‐A68.9,A69.2‐A69.5,A75‐A75.9,A77‐A79.9,A92‐A94.0,A96‐A96.9,A98‐A98.8,B58,B59‐B60.8,B68‐B68.9,B70‐B72.0,B74.3‐B75,B78‐B78.9,B80‐B81.8,B83‐B83.8,P37.1 |
| Maternal disorders | N96,N98‐N98.9,O00‐O07.9,O09‐O16.9,O20‐O26.93,O28‐O36.93,O40‐O48.1,O60‐O77.9,O80‐O92.79,O96‐O99.91 |
| Maternal hemorrhage | O20‐O20.9,O43.2‐O43.239,O44‐O46.93,O67‐O67.9,O70,O72‐O72.3 |
| Maternal sepsis and other maternal infections | O23‐O23.93,O85‐O86.89,O91‐O91.23 |
| Maternal hypertensive disorders | O10‐O16.9 |
| Maternal obstructed labor | O32‐O33.9,O64‐O66.9,O71‐O71.9 |
| Maternal abortive outcome | N96,O00‐O07.9 |
| Indirect maternal deaths | O24‐O25.3,O98‐O99.91 |
| Late maternal deaths | O96‐O97.9 |
| Maternal deaths aggravated by HIV/AIDS | N98‐N98.9,O09‐O09.93,O21‐O22.93,O26‐O26.93,O28‐O31.8,O34‐O36.93,O40‐O43.199,O43.8‐O43.93,O47‐ O48.1,O60‐O63.9,O68‐O69.9,O70.0‐O70.9,O73‐O77.9,O80‐O84.9,O87‐O90.9,O92‐O92.79 |
| Neonatal disorders | P00‐P03.1,P03.6‐P05.9,P07‐P15.9,P19‐P22.9,P24‐P29.9,P36‐P36.9,P38‐P39.9,P50‐P61.9,P70,P70.3‐P72.9,P74‐P78.9,P80‐P81.9,P83‐P84,P90‐P94.9,P96,P96.3,P96.4,P96.8‐P96.89 |
| Neonatal preterm birth complications | P01.0,P01.1,P07‐P07.39,P22‐P22.9,P25‐P28.9,P61.2,P77‐P77.9 |
| Neonatal encephalopathy due to birth asphyxia and trauma | P01.7‐P03.1,P03.6‐P03.9,P10‐P15.9,P20‐P21.9,P24‐P24.9,P90‐P91.9 |
| Neonatal sepsis and other neonatal infections | P36‐P36.9,P38‐P39.9 |
| Hemolytic disease and other neonatal jaundice | P55‐P59.9 |
| Other neonatal disorders | P00‐P01,P01.2‐P01.6,P04‐P05.9,P08‐P09,P19‐P19.9,P29‐P29.9,P50‐P54.9,P60‐P61.1,P61.3‐P61.9,P70,P70.3‐ P72.9,P74‐P76.9,P78‐P78.9,P80‐P81.9,P83‐P84,P92‐P94.9,P96,P96.3,P96.4,P96.8‐P96.89 |
| Nutritional deficiencies | D50‐D52.0,D52.8‐D53.9,D62‐D63.8,D64.1‐D64.3,D64.8‐D64.9,D69.9,E00‐E02,E40‐E46.9,E51‐E61.9,E63‐E64.0,E64.2‐E64.9,M12.1‐M12.19 |
| Protein‐energy malnutrition | E40‐E46.9,E64.0 |
| Iodine deficiency | E00‐E02 |
| Iron‐deficiency anemia | D50‐D50.9,D62‐D63.8,D64.1‐D64.3,D64.8‐D64.9,D69.9 |
| Other nutritional deficiencies | D51‐D52.0,D52.8‐D53.9,E51‐E61.9,E63‐E64,E64.2,E64.3,M12.1‐M12.19 |
| Other communicable, maternal, neonatal, and nutritional diseases | A20‐A28.9,A31,A31.0,A31.8‐A32.9,A38‐A38.9,A42‐A44.9,A48.1,A48.2,A48.4‐A48.52,A49.1,A50‐A58,A63‐A63.8,A65 A67.9,A69‐A69.1,A69.8‐A70,A74,A74.8‐A74.9,A80‐A81.9,A88‐A89.9,B00‐B00.9,B03,B04,B06‐B06.9,B10‐ B10.89,B15‐B17.2,B19.1‐B19.21,B25‐B27.99,B29.4,B33‐B33.8,B37‐B37.2,B37.5‐B37.89,B38‐B44.9,B46‐B49.9,B58.0 B58.9,B63,B91,B94.2,B95‐B95.5,G14,G14.6,I00,I02,I02.9,J02.0,J03.0‐J03.01,K67.0‐ K67.2,K67.8,K74.7,K74.8,M03.1,M49.1,M73.0,M73.1,M89.6‐M89.69,N70‐N71.9,N73‐N74.8,P35‐ |
| Sexually transmitted diseases excluding HIV | A50‐A58,A63‐A63.8,B63,K67.0‐K67.2,M03.1,M73.0,M73.1,N70‐N71.9,N73‐N74.8 |
| Syphilis | A50‐A53.9,K67.2,M03.1,M73.1 |
| Chlamydial infection | A55‐A56.8,K67.0 |
| Gonococcal infection | A54‐A54.9,K67.1,M73.0 |
| Other sexually transmitted diseases | A57‐A58,A63‐A63.8 |

| **Table S2. List of International Classification of Diseases (ICD) codes mapped to the Global Burden of Disease cause list for causes of death** | |
| --- | --- |
| **Cause** | **ICD10 Code** |
| Hepatitis | B15‐B17.2,B19.1‐B19.21,B94.2,P35.3 |
| Acute hepatitis A | B15‐B15.9 |
| Acute hepatitis B | B16‐B17.0,B19.1‐B19.11,P35.3 |
| Acute hepatitis C | B17.1‐B17.11,B19.2‐B19.21 |
| Acute hepatitis E | B17.2 |
| Other infectious diseases | A20‐A28.9,A31,A31.0,A31.8‐A32.9,A38‐A38.9,A42‐A44.9,A48.1,A48.2,A48.4‐A48.52,A49.1,A65‐A67.9,A69‐ A69.1,A69.8‐A70,A74,A74.8‐A74.9,A80‐A81.9,A88‐A89.9,B00‐B00.9,B03,B04,B06‐B06.9,B10‐B10.89,B25‐ B27.99,B29.4,B33‐B33.8,B37‐B37.2,B37.5‐B37.89,B38‐B44.9,B46‐B49.9,B58.0‐B58.9,B91,B95‐ B95.5,G14,G14.6,I00,I02,I02.9,J02.0,J03.0‐J03.01,K67.8,K74.7,K74.8,M49.1,M89.6‐M89.69,P35‐P35 2 P35 9 P37 P37 2 P37 5‐P37 9 |
| Non‐communicable diseases | A31.1,A31.2,A46,A46.0,B18‐B18.9,B37.3‐B37.49,B37.9,C00‐C13.9,C15‐C25.9,C30‐C34.92,C37‐C38.8,C40‐C41.9,C43 C45.9,C47‐C54.9,C56‐C57.8,C58,C58.0,C60‐C63.8,C64‐C67.9,C68.0‐C68.8,C69‐C75.8,C81‐C86.6,C88‐C97.9,D00.00‐ D00.2,D01.0‐D01.3,D02.0‐D02.3,D03‐D06.9,D07.0‐D07.2,D07.4,D07.5,D09.0,D09.2‐D09.8,D10.0‐D10.7,D11‐ D12.9,D13.0‐D13.7,D14.0‐D14.32,D15‐D16.9,D22‐D25.9,D26.0,D26.1,D27‐D27.9,D28.0‐D28.7,D29.0‐D29.8,D30.0‐ D30.8,D31‐D36,D36.1‐D36.7,D37.01‐D37.5,D38.0‐D38.5,D39.1‐D39.8,D40.0‐D40.8,D41.0‐D41.8,D42‐D43.9,D44.0‐ D44.8,D45‐D47.9,D48.0‐D48.62,D49.2‐D49.4,D49.6,D49.81,D52.1,D55‐D58.9,D59.0‐D59.3,D59.5,D59.6,D60‐ D61.9,D64.0,D64.4,D66‐D69.8,D70‐D75.89,D76‐D78.89,D80‐D83.9,D84.0‐D84.8,D86‐D86.9,D89‐D89.3,E03‐ E07.1,E09‐E16.9,E20‐E34.8,E36‐E36.8,E65‐E68,E70‐E85.29,E87.71,E88‐E89.9,F00‐F03.91,F06.2,F10‐F11.99,F13‐ F16.99,F18‐F23.9,F25‐F29.9,F50.0‐F50.1,G10‐G13.8,G20‐G26.0,G30‐G31.9,G35‐G37.9,G40‐G41.9,G45‐ G46.8,G47.3‐G47.39,G61‐G61.9,G70‐G73.7,G90‐G90.9,G93.7,G95‐G95.9,G97‐G97.9,I01‐I01.9,I02.0,I05‐I09.9,I11‐ I13.9,I20‐I25.9,I27.1,I28‐I28.8,I30‐I31.1,I31.8,I31.9,I33‐I42.9,I47‐I48.92,I51.0‐I51.6,I60‐I61.9,I62.0‐I62.03,I63‐ I63.9,I65‐I66.9,I67.0‐I67.3,I67.5‐I67.7,I69.0‐I69.198,I69.20‐I69.398,I70.2‐I70.8,I71‐I78.9,I80‐ I89.9,I91.9,I95.2,I95.3,I97‐I98.2,I98.9,J30‐J35.9,J37‐J47.9,J60‐J63.8,J65‐J68.9,J70‐J70.9,J82,J84‐J84.9,J91‐J92.9,J95‐ J95.9,K20‐K22.9,K25‐K29.91,K31‐K31.89,K35‐K38.9,K40‐K46.9,K50‐K52.9,K55‐K57.93,K58.0‐K62.9,K63.5,K64‐ K64.9,K66.8,K67,K68‐K68.9,K70‐K70.9,K71.3‐K71.51,K71.7,K72.1‐K74.69,K74.9,K75.2‐K77.8,K80‐K83.9,K85‐ K86.9,K90‐K92,K92.8‐K92.89,K94‐K95.89,L00‐L05.92,L08‐L08.9,L10‐L14.0,L51‐L51.9,L88‐L89.95,L93‐L93.2,L97‐ L98.499,M00‐M03.0,M03.2,M03.6,M05‐M09.8,M30‐M36.8,M40‐M43.19,M65‐M65.08,M71.0‐M71.19,M86‐ M87.19,M88‐M89.09,M89.5‐M89.59,M89.7‐M89.9,N00‐N08.8,N10‐N12.9,N14‐N16.8,N18‐N18.9,N20‐N23.0,N25‐ N32.0,N32.3,N32.4,N34‐N34.3,N36‐N36.9,N39‐N39.2,N41‐N41.9,N44‐N45.9,N49‐N51.8,N65‐ N65.1,N72,N72.0,N75‐N77.8,N80‐N81.9,N83‐N83.9,N84.0,N84.1,N87‐N87.9,N99‐N99.9,P03.2‐P03.5,P70.0‐ P70.2,P96.0‐P96.2,P96.5,Q00‐Q07.9,Q10.4‐Q18.9,Q20‐Q28.9,Q30‐Q36,Q37‐Q45.9,Q50‐Q87.89,Q89‐Q89.8,Q90‐ Q93.9,Q95‐Q95.9,Q97‐Q97.9,Q99‐Q99.8,R50.2,R50.82,R50.83,R73‐R73.9,R78.0‐R78.5,R95,X45‐X45.9,X49‐X49.9 |

| **Table S2. List of International Classification of Diseases (ICD) codes mapped to the Global Burden of Disease cause list for causes of death** | |
| --- | --- |
| **Cause** | **ICD10 Code** |
| Neoplasms | C00‐C13.9,C15‐C25.9,C30‐C34.92,C37‐C38.8,C40‐C41.9,C43‐C45.9,C47‐C54.9,C56‐C57.8,C58,C58.0,C60‐C63.8,C64‐ C67.9,C68.0‐C68.8,C69‐C75.8,C81‐C86.6,C88‐C97.9,D00.00‐D00.2,D01.0‐D01.3,D02.0‐D02.3,D03‐D06.9,D07.0‐ D07.2,D07.4,D07.5,D09.0,D09.3‐D09.8,D10.0‐D10.7,D11‐D12.9,D13.0‐D13.7,D14.0‐D14.32,D15‐D16.9,D22‐ D25.9,D26.0,D26.1,D27‐D27.9,D28.0‐D28.7,D29.0‐D29.8,D30.0‐D30.8,D31‐D36,D36.1‐D36.7,D37.01‐D37.5,D38.0‐ D38.5,D39.1‐D39.8,D40.0‐D40.8,D41.0‐D41.8,D42‐D43.9,D44.0‐D44.8,D45‐D47.9,D48.0‐D48.62,D49.2‐ D49.4,D49.6,D49.81,K31.7,K62.0,K62.1,K63.5,N84.0,N84.1,N87‐N87.9 |
| Esophageal cancer | C15‐C15.9,D00.1,D13.0 |
| Stomach cancer | C16‐C16.9,D00.2,D13.1,D37.1 |
| Liver cancer | C22‐C22.9,D13.4 |
| Larynx cancer | C32‐C32.9,D02.0,D14.1,D38.0 |
| Tracheal, bronchus and lung cancers | C33‐C34.92,D02.1‐D02.3,D14.2‐D14.32,D38.1 |
| Breast cancer | C50‐C50.929,D05‐D05.92,D24‐D24.9,D48.6‐D48.62,D49.3 |
| Cervical cancer | C53‐C53.9,D06‐D06.9,D26.0,D26.1,N84.1 |
| Uterine cancer | C54‐C54.9,D25‐D25.9,N84.0,N87‐N87.9 |
| Prostate cancer | C61‐C61.9,D29.1,D40.0 |
| Colon and rectum cancer | C18‐C20.0,C20.9‐C21.8,D01.0‐D01.3,D12‐D12.9,D37.3‐D37.5,K62.0,K62.1,K63.5 |
| Lip and oral cavity cancer | C00‐C08.9,D00.00‐D00.07,D10.0‐D10.5,D11‐D11.9,D37.01‐D37.04,D37.09 |
| Nasopharynx cancer | C11‐C11.9,D00.08,D10.6,D37.05 |
| Other pharynx cancer | C09‐C10.9,C12‐C13.9,D10.7 |
| Gallbladder and biliary tract cancer | C20.8,C23‐C24.9,D13.5 |
| Pancreatic cancer | C25‐C25.9,D13.6,D13.7 |
| Malignant skin melanoma | C43‐C43.9,C4A,D03‐D03.9,D22‐D23.9,D48.5 |
| Non‐melanoma skin cancer | C44‐C44.99,D04‐D04.9,D49.2 |
| Ovarian cancer | C56‐C56.9,D27‐D27.9,D39.1‐D39.12 |
| Testicular cancer | C62‐C62.92,D29.2‐D29.8,D40.1‐D40.8 |
| Kidney cancer | C64‐C65.9,D30.0‐D30.12,D41.0‐D41.12 |
| Bladder cancer | C67‐C67.9,D09.0,D30.3,D41.4‐D41.8,D49.4 |
| Brain and nervous system cancer | C70‐C72.9 |
| Thyroid cancer | C73‐C73.9,D09.3‐D09.8,D34‐D34.9,D44.0 |
| Mesothelioma | C45‐C45.9 |
| Hodgkin lymphoma | C81‐C81.99 |
| Non‐Hodgkin lymphoma | C82‐C86.6,C96‐C97.9 |
| Multiple myeloma | C88‐C90.32 |
| Leukemia | C91‐C95.92 |

| **Table S2. List of International Classification of Diseases (ICD) codes mapped to the Global Burden of Disease cause list for causes of death** | |
| --- | --- |
| **Cause** | **ICD10 Code** |
| Other neoplasms | C17‐C17.9,C30‐C31.9,C37‐C38.8,C40‐C41.9,C47‐C49.9,C51‐C52.9,C57‐C57.8,C58,C58.0,C60‐C60.9,C63‐C63.8,C66‐ C66.9,C68.0‐C68.8,C69‐C69.92,C74‐C75.8,D07.0‐D07.2,D07.4,D07.5,D13.2‐D13.39,D14.0,D15‐D16.9,D28.0‐ D28.7,D29.0,D30.2‐D30.22,D30.4‐D30.8,D31‐D33.9,D35‐D36,D36.1‐D36.7,D37.2,D38.2‐D38.5,D39.2‐D39.8,D41.2‐ D41.3,D42‐D43.9,D44.1‐D44.8,D45‐D47.9,D48.0‐D48.4,D49.6,D49.81,K31.7 |
| Cardiovascular diseases | G45‐G46.8,I01‐I01.9,I02.0,I05‐I09.9,I11‐I11.9,I20‐I25.9,I28‐I28.8,I30‐I31.1,I31.8,I31.9,I33‐I42.9,I47‐I48.92,I51.0‐ I51.6,I60‐I61.9,I62.0‐I62.03,I63‐I63.9,I65‐I66.9,I67.0‐I67.3,I67.5‐I67.7,I69.0‐I69.198,I69.20‐I69.398,I70.2‐I70.8,I71‐ I78.9,I80‐I83.93,I86‐I89.9,I91.9 |
| Rheumatic heart disease | I01‐I01.9,I02.0,I05‐I09.9 |
| Ischemic heart disease | I20‐I25.9 |
| Cerebrovascular disease | G45‐G46.8,I60‐I61.9,I62.0‐I62.03,I63‐I63.9,I65‐I66.9,I67.0‐I67.3,I67.5‐I67.7,I69.0‐I69.198,I69.20‐I69.398 |
| Ischemic stroke | G45‐G46.8,I63‐I63.9,I65‐I66.9,I67.2,I67.3,I67.5,I67.6,I69.3‐I69.398 |
| Hemorrhagic stroke | I60‐I61.9,I62.0‐I62.03,I67.0,I67.1,I67.7,I69.0‐I69.198,I69.20‐I69.298 |
| Hypertensive heart disease | I11‐I11.9 |
| Cardiomyopathy and myocarditis | I40‐I42.9,I51.4‐I51.6 |
| Atrial fibrillation and flutter | I48‐I48.92 |
| Aortic aneurysm | I71‐I71.9 |
| Peripheral vascular disease | I70.2‐I70.799,I73‐I73.9 |
| Endocarditis | I33‐I33.9,I39‐I39.9 |
| Other cardiovascular and circulatory diseases | I28‐I28.8,I30‐I31.1,I31.8,I31.9,I34‐I38.9,I47‐I47.9,I51.0‐I51.3,I70.8,I72‐I72.9,I74‐I78.9,I80‐I83.93,I86‐I89.9,I91.9 |
| Chronic respiratory diseases | D86‐D86.2,D86.89,D86.9,G47.3‐G47.39,J30‐J35.9,J37‐J47.9,J60‐J63.8,J65‐J68.9,J70‐J70.1,J70.8,J70.9,J82,J84‐J84.9,J91‐J92.9 |
| Chronic obstructive pulmonary disease | J40‐J44.9,J47‐J47.9 |
| Pneumoconiosis | J60‐J63.8,J65,J65.0,J92.0 |
| Silicosis | J62‐J62.9 |
| Asbestosis | J61,J61.0,J92.0 |
| Coal workers pneumoconiosis | J60,J60.0 |
| Other pneumoconiosis | J63‐J63.8,J65,J65.0 |
| Asthma | J45‐J46.9 |
| Interstitial lung disease and pulmonary sarcoidosis | D86‐D86.2,D86.89,D86.9,J84‐J84.9 |
| Other chronic respiratory diseases | G47.3‐G47.39,J30‐J35.9,J37‐J39.9,J66‐J68.9,J70‐J70.1,J70.8,J70.9,J82,J91‐J92,J92.9 |
| Cirrhosis | B18‐B18.9,D09.2‐D09.22,I85‐I85.9,K70‐K70.9,K71.3‐K71.51,K71.7,K72.1‐K74.69,K74.9,K75.8‐K76.0,K76.6,K76.7,K76.9 |

| **Table S2. List of International Classification of Diseases (ICD) codes mapped to the Global Burden of Disease cause list for causes of death** | |
| --- | --- |
| **Cause** | **ICD10 Code** |
| Cirrhosis due to alcohol use | D09.2‐D09.22 |
| Digestive diseases | I84‐I84.9,K20‐K22.9,K25‐K29.91,K31‐K31.6,K31.8‐K31.89,K35‐K38.9,K40‐K42.9,K44‐K46.9,K50‐K52.9,K55‐ K57.93,K58.0‐K62,K62.2‐K62.6,K62.8‐K62.9,K64‐K64.9,K66.8,K67,K68‐K68.9,K75.2‐K75.4,K76.1‐K76.5,K76.8‐ K76.89,K77‐K77.8,K80‐K83.9,K85‐K86.9,K90‐K90.9,K92.8‐K92.89,M09.1 |
| Peptic ulcer disease | K25‐K28.9,K31,K31.1‐K31.6,K31.8,K31.82‐K31.89 |
| Gastritis and duodenitis | K29‐K29.91 |
| Appendicitis | K35‐K37.9,K38.3‐K38.9 |
| Paralytic ileus and intestinal obstruction | K56‐K56.9 |
| Inguinal, femoral, and abdominal hernia | K40‐K42.9,K44‐K46.9 |
| Inflammatory bowel disease | K50‐K52.9,K58.0,M09.1 |
| Vascular intestinal disorders | K55‐K55.9 |
| Gallbladder and biliary diseases | K80‐K83.9 |
| Pancreatitis | K85‐K86.9 |
| Other digestive diseases | I84‐I84.9,K20‐K22.9,K31.0,K31.81‐K31.819,K38‐K38.2,K57‐K57.93,K58.9‐K62,K62.2‐K62.6,K62.8‐K62.9,K64‐ K64.9,K66.8,K67,K68‐K68.9,K75.2‐K75.4,K76.1‐K76.5,K76.8‐K76.89,K77‐K77.8,K90‐K90.9,K92.8‐K92.89 |
| Neurological disorders | F00‐F03.91,G10‐G13.8,G20‐G21.0,G21.2‐G24,G24.1‐G25.0,G25.2,G25.3,G25.5,G25.8‐G26.0,G30‐G31.9,G35‐G37.9,G40‐G41.9,G61‐G61.9,G70‐G72,G72.2‐G73.7,G90‐G90.9,G95‐G95.9,M33‐M33.99 |
| Alzheimer disease and other dementias | F00‐F03.91,G30‐G31.9 |
| Parkinson disease | G20‐G21.0,G21.2‐G22.0 |
| Epilepsy | G40‐G41.9 |
| Multiple sclerosis | G35‐G35.9 |
| Other neurological disorders | G10‐G13.8,G23‐G24,G24.1‐G25.0,G25.2,G25.3,G25.5,G25.8‐G26.0,G36‐G37.9,G61‐G61.9,G70‐G72,G72.2‐G73.7,G90‐G90.9,G95‐G95.9,M33‐M33.99 |
| Mental and substance use disorders | F06.2,F10‐F11.99,F13‐F16.99,F18‐F23.9,F25‐F29.9,F50.0‐F50.1,G72.1,P96.1,Q86.0,R78.0‐R78.5,X45‐X45.9,X49‐X49.9 |
| Schizophrenia | F06.2,F20‐F23.9,F25‐F29.9 |
| Alcohol use disorders | F10‐F10.99,G72.1,Q86.0,R78.0,X45‐X45.9 |
| Drug use disorders | F11‐F11.99,F13‐F16.99,F18‐F19.99,P96.1,R78.1‐R78.5,X49‐X49.9 |
| Opioid use disorders | F11‐F11.99,P96.1,R78.1 |
| Cocaine use disorders | F14‐F14.99,R78.2 |
| Amphetamine use disorders | F15‐F15.99 |
| Other drug use disorders | F13‐F13.99,F16‐F16.99,F18‐F19.99,R78.3‐R78.5,X49‐X49.9 |
| Eating disorders | F50.0‐F50.1 |
| Anorexia nervosa | F50.0‐F50.1 |

| **Table S2. List of International Classification of Diseases (ICD) codes mapped to the Global Burden of Disease cause list for causes of death** | |
| --- | --- |
| **Cause** | **ICD10 Code** |
| Diabetes, urogenital, blood, and endocrine diseases | B37.3‐B37.49,B37.9,D52.1,D55‐D58.9,D59.0‐D59.3,D59.5,D59.6,D60‐D61.9,D64.0,D64.4,D66‐D69.8,D70‐ D75.89,D76‐D78.89,D80‐D83.9,D84.0‐D84.8,D86.3‐D86.87,D89‐D89.3,E03‐E07.1,E09‐E16.9,E20‐E34.8,E36‐ E36.8,E65‐E68,E70‐E85.29,E87.71,E88‐E89.9,G21.1‐G21.19,G24.0‐G24.09,G25.1,G25.4,G25.6‐ G25.79,G72.0,G93.7,G97‐G97.9,I12‐I13.9,I95.2,I95.3,I97‐I98.2,I98.9,J70.2‐J70.5,J95‐J95.9,K43‐K43.9,K62.7,K91‐ K92,K94‐K95.89,M87.1‐M87.19,N00‐N08.8,N10‐N12.9,N14‐N16.8,N18‐N18.9,N20‐N23.0,N25‐ N32.0,N32.3,N32.4,N34‐N34.3,N36‐N36.9,N39‐N39.2,N41‐N41.9,N44‐N45.9,N49‐N51.8,N65‐ N65.1,N72,N72.0,N75‐N77.8,N80‐N81.9,N83‐N83.9,N99‐N99.9,P03.2‐P03.5,P70.0‐ |
| Diabetes mellitus | E10‐E10.11,E10.3‐E11.1,E11.3‐E12.1,E12.3‐E13.11,E13.3‐E14.1,E14.3‐E14.9,P70.0‐P70.2,R73‐R73.9 |
| Acute glomerulonephritis | N00‐N01.9 |
| Chronic kidney disease | E10.2‐E10.29,E11.2‐E11.29,E12.2,E13.2‐E13.29,E14.2,I12‐I13.9,N02‐N08.8,N15.0,N18‐N18.9 |
| Chronic kidney disease due to diabetes mellitus | E10.2‐E10.29,E11.2‐E11.29,E12.2,E13.2‐E13.29,E14.2 |
| Chronic kidney disease due to hypertension | I12‐I13.9 |
| Chronic kidney disease due to glomerulonephritis | N03‐N06.9 |
| Chronic kidney disease due to other causes | N02‐N02.9,N07‐N08.8,N15.0 |
| Urinary diseases and male infertility | N10‐N12.9,N15,N15.1‐N16.8,N20‐N23.0,N25‐N32.0,N32.3,N32.4,N34‐N34.3,N36‐N36.9,N39‐N39.2,N41‐N41.9,N44‐N45.9,N49‐N51.8 |
| Interstitial nephritis and urinary tract infections | N10‐N12.9,N15,N15.1‐N16.8,N30‐N30.91,N34‐N34.3,N39.0‐N39.2 |
| Urolithiasis | N20‐N23.0 |
| Other urinary diseases | N25‐N29.8,N31‐N32.0,N32.3,N32.4,N36‐N36.9,N39,N41‐N41.9,N44‐N45.9,N49‐N51.8 |
| Gynecological diseases | B37.3‐B37.49,B37.9,E28.2,N72,N72.0,N75‐N77.8,N80‐N81.9,N83‐N83.9 |
| Polycystic ovarian syndrome | E28.2 |
| Endometriosis | N80‐N80.9 |
| Genital prolapse | N81‐N81.9 |
| Other gynecological diseases | B37.3‐B37.49,B37.9,N72,N72.0,N75‐N77.8,N83‐N83.9 |
| Hemoglobinopathies and hemolytic anemias | D55‐D58.9,D59.1,D59.3,D59.5,D60‐D61.09,D61.2‐D61.9,D64.0,D64.4 |
| Thalassemias | D56‐D56.9 |
| Sickle cell disorders | D57‐D57.819 |
| G6PD deficiency | D55‐D55.9 |
| Other hemoglobinopathies and hemolytic anemias | D58‐D58.9,D59.1,D59.3,D59.5,D60‐D61.09,D61.2‐D61.9,D64.0,D64.4 |
| Endocrine, metabolic, blood, and immune disorders | D52.1,D59.0,D59.2,D59.6,D61.1,D66‐D69.8,D70‐D75.89,D76‐D78.89,D80‐D83.9,D84.0‐D84.8,D86.3‐D86.87,D89‐ D89.3,E03‐E07.1,E09‐E09.9,E15‐E16.9,E20‐E28.1,E28.3‐E34.8,E36‐E36.8,E65‐E68,E70‐E85.29,E87.71,E88‐ E89.9,G21.1‐G21.19,G24.0‐G24.09,G25.1,G25.4,G25.6‐G25.79,G72.0,G93.7,G97‐G97.9,I95.2,I95.3,I97‐ I98.2,I98.9,J70.2‐J70.5,J95‐J95.9,K43‐K43.9,K62.7,K91‐K92,K94‐K95.89,M87.1‐M87.19,N14‐N14.4,N65‐N65.1,N99‐N99 9 P03 2‐P03 5 P96 2 P96 5 R50 2 R50 82 R50 8 |
| Musculoskeletal disorders | I27.1,L93‐L93.2,M00‐M03.0,M03.2,M03.6,M05‐M09.0,M09.2,M09.8,M30‐M32.9,M34‐M36.8,M40‐M43.19,M65‐M65.08,M71.0‐M71.19,M86‐M87.09,M88‐M89.09,M89.5‐M89.59,M89.7‐M89.9 |
| Rheumatoid arthritis | M05‐M06.9,M08.0‐M08.89 |

| **Table S2. List of International Classification of Diseases (ICD) codes mapped to the Global Burden of Disease cause list for causes of death** | |
| --- | --- |
| **Cause** | **ICD10 Code** |
| Other musculoskeletal disorders | I27.1,L93‐L93.2,M00‐M03.0,M03.2,M03.6,M07‐M08,M08.9‐M09.0,M09.2,M09.8,M30‐M32.9,M34‐M36.8,M40‐ M43.19,M65‐M65.08,M71.0‐M71.19,M86‐M87.09,M88‐M89.09,M89.5‐M89.59,M89.7‐M89.9 |
| Other non‐communicable diseases | A31.1,A31.2,A46,A46.0,L00‐L05.92,L08‐L08.9,L10‐L14.0,L51‐L51.9,L88‐L89.95,L97‐L98.499,P96.0,Q00‐Q07.9,Q10.4 Q18.9,Q20‐Q28.9,Q30‐Q36,Q37‐Q45.9,Q50‐Q86,Q86.1‐Q87.89,Q89‐Q89.8,Q90‐Q93.9,Q95‐Q95.9,Q97‐Q97.9,Q99‐Q99.8,R95 |
| Congenital anomalies | P96.0,Q00‐Q07.9,Q10.4‐Q18.9,Q20‐Q28.9,Q30‐Q36,Q37‐Q45.9,Q50‐Q86,Q86.1‐Q87.89,Q89‐Q89.8,Q90‐Q93.9,Q95‐Q95.9,Q97‐Q97.9,Q99‐Q99.8 |
| Neural tube defects | Q00‐Q01.9,Q05‐Q05.9 |
| Congenital heart anomalies | Q20‐Q28.9 |
| Cleft lip and cleft palate | Q35‐Q36,Q37‐Q37.9 |
| Down syndrome | Q90‐Q90.9 |
| Chromosomal unbalanced rearrangements | Q91‐Q93.9,Q95‐Q95.9,Q97‐Q97.9,Q99‐Q99.8 |
| Other congenital anomalies | P96.0,Q02‐Q04.9,Q06‐Q07.9,Q10.4‐Q18.9,Q30‐Q34.9,Q38‐Q45.9,Q50‐Q86,Q86.1‐Q87.89,Q89‐Q89.8 |
| Skin and subcutaneous diseases | A31.1,A31.2,A46,A46.0,L00‐L05.92,L08‐L08.9,L10‐L14.0,L51‐L51.9,L88‐L89.95,L97‐L98.499 |
| Cellulitis | L03‐L04 |
| Abscess, impetigo, and other bacterial skin diseases | A31.1,A31.2,A46,A46.0,L00‐L02.93,L04.0‐L05.92,L08‐L08.9,L88,L97‐L98.499 |
| Decubitus ulcer | L89‐L89.95 |
| Other skin and subcutaneous diseases | L10‐L14.0,L51‐L51.9 |
| Sudden infant death syndrome | R95 |
| Injuries | V00‐V86.99,V87.2,V87.3,V88.2,V88.3,V90,V90.0,V90.01‐V98.8,W00‐W46.2,W49‐W62.9,W64‐W70.9,W73‐ W81.9,W83‐W94.9,W97.9,W99‐X06.9,X08‐X40.9,X43‐X43.9,X46‐X48.9,X50‐X54.9,X57‐X58.9,X60‐Y08.9,Y35‐ Y84.9,Y87.1,Y88‐Y88.3,Y89.0,Y89.1 |
| Transport injuries | V00‐V86.99,V87.2,V87.3,V88.2,V88.3,V91‐V91.9,V93‐V98.8 |
| Road injuries | V01‐V04.99,V06‐V80.929,V82‐V82.9,V87.2,V87.3 |

| **Table S2. List of International Classification of Diseases (ICD) codes mapped to the Global Burden of Disease cause list for causes of death** | |
| --- | --- |
| **Cause** | **ICD10 Code** |
| Pedestrian road injuries | V01‐V04.99,V06‐V09.9 |
| Cyclist road injuries | V10‐V19.9 |
| Motorcyclist road injuries | V20‐V29.9 |
| Motor vehicle road injuries | V30‐V79.9,V87.2,V87.3 |
| Other road injuries | V80‐V80.929,V82‐V82.9 |
| Other transport injuries | V00‐V00.898,V05‐V05.99,V81‐V81.9,V83‐V86.99,V88.2,V88.3,V91‐V91.9,V93‐V98.8 |
| Unintentional injuries | V90,V90.0,V90.01‐V90.9,V92‐V92.9,W00‐W46.2,W49‐W62.9,W64‐W70.9,W73‐W81.9,W83‐W94.9,W97.9,W99‐ X06.9,X08‐X29.9,X40‐X40.9,X43‐X43.9,X46‐X48.9,X50‐X54.9,X57‐X58.9,Y38.9‐Y84.9,Y88‐Y88.3 |
| Falls | W00‐W19.9 |
| Drowning | V90,V90.0,V90.01‐V90.9,V92‐V92.9,W65‐W70.9,W73‐W74.9 |
| Fire, heat, and hot substances | X00‐X06.9,X08‐X19.9,X20.5 |
| Poisonings | X40‐X40.9,X43‐X43.9,X46‐X48.9 |
| Exposure to mechanical forces | W20‐W38.9,W40‐W43.9,W45.0‐W45.2,W46‐W46.2,W49‐W52,W52.3,W75‐W76.9 |
| Unintentional firearm injuries | W32‐W34.9 |
| Unintentional suffocation | W75‐W76.9 |
| Other exposure to mechanical forces | W20‐W31.9,W35‐W38.9,W40‐W43.9,W45.0‐W45.2,W46‐W46.2,W49‐W52,W52.3 |
| Adverse effects of medical treatment | Y38.9‐Y84.9,Y88‐Y88.3 |
| Animal contact | W52.0‐W52.2,W52.4‐W62.9,W64‐W64.9,X20‐X20.4,X20.6‐X29.9 |
| Venomous animal contact | X20‐X20.4,X20.6‐X29.9 |
| Non‐venomous animal contact | W52.0‐W52.2,W52.4‐W62.9,W64‐W64.9 |
| Foreign body | W44‐W45,W45.3‐W45.9,W78‐W80.9,W83‐W84.9 |
| Pulmonary aspiration and foreign body in airway | W78‐W80.9,W83‐W84.9 |
| Foreign body in other body part | W44‐W45,W45.3‐W45.9 |
| Other unintentional injuries | W39‐W39.9,W77‐W77.9,W81‐W81.9,W85‐W94.9,W97.9,W99‐W99.9,X50‐X54.9,X57‐X58.9 |
| Self‐harm and interpersonal violence | X60‐Y08.9,Y87.1 |

| **Table S2. List of International Classification of Diseases (ICD) codes mapped to the Global Burden of Disease cause list for causes of death** | |
| --- | --- |
| **Cause** | **ICD10 Code** |
| Self‐harm | X60‐X84.9 |
| Interpersonal violence | X85‐Y08.9,Y87.1 |
| Assault by firearm | X93‐X94.0,X94.3‐X94.7,X94.9‐X95.9,X96.5 |
| Assault by sharp object | X99‐X99.9 |
| Assault by other means | X85‐X92.9,X94.1,X94.2,X94.8,X96‐X96.4,X96.6‐X98.9,Y00‐Y08.9,Y87.1 |
| Forces of nature, war, and legal intervention | X30‐X39.9,Y35‐Y38.893,Y89.0,Y89.1 |
| Exposure to forces of nature | X30‐X39.9 |
| Collective violence and legal intervention | Y35‐Y38.893,Y89.0,Y89.1 |
| Unspecified Code | U00‐U04,U05‐U99 |
| Still born | P95‐P95.9 |
| Garbage Code | A01,A14.9,A29‐A30.9,A40‐A41.9,A45,A45.9,A47‐A48.0,A48.3,A48.8‐A49.02,A49.2‐A49.9,A59‐A62,A64,A64.0,A71‐ A73,A74.0,A76,A97,A99,A99.0,B07‐B09,B11‐B14,B17.8,B17.9,B19,B19.0,B19.9,B28,B29,B30‐B32.4,B34‐ B36.9,B55,B55.1‐B55.9,B61,B62,B64,B66‐B66.9,B73‐B74.2,B76‐B76.9,B79,B82‐B82.9,B83.9‐B89,B92‐ B94.0,B94.8,B94.9,B95.6‐B99.9,C14‐C14.9,C26‐C29,C35,C36,C39‐C39.9,C42,C46‐C46.9,C55‐ C55.9,C57.9,C59,C63.9,C68,C68.9,C75.9‐C80.9,C87,C98‐D00.0,D01,D01.4‐D02,D02.4,D02.9,D07,D07.3‐ D07.39,D07.6‐D09,D09.1‐D09.19,D09.9,D10,D10.9,D13,D13.9,D14,D14.4,D17‐ D21.9,D26,D26.7,D26.9,D28,D28.9,D29,D29.9,D30,D30.9,D36.0,D36.9‐D37.0,D37.6‐D38,D38.6‐ D39.0,D39.9,D40,D40.9,D41,D41.9,D44,D44.9,D48,D48.7‐ D49.1,D49.5,D49.7,D49.8,D49.89,D49.9,D54,D59,D59.4,D59.8,D59.9,D64,D65‐ D65.9,D75.9,D79,D84,D84.9,D85,D87,D88,D89.8‐D99,E07.8‐E08.9,E17‐E19,E34.9‐E35.8,E37‐E39,E47‐ E50.9,E62,E64.1,E69,E85.3‐E87.70,E87.79‐E87.99,E90‐E99.9,F04‐F06.1,F06.3‐F07.0,F07.2‐F09.9,F12‐F12.99,F17‐ F17.9,F24,F30‐F50,F50.2‐G00,G00.9‐G02.8,G03.9,G06‐G09.0,G15‐G19,G27‐G29,G32‐G34,G38,G39,G42‐ G44.89,G47‐G47.29,G47.4‐G60.9,G62‐G69,G74‐G89.4,G91‐G93.6,G93.8‐G94.8,G96‐G96.9,G98‐H69.93,H71‐ H99,I00.0,I03,I04,I10‐I10.9,I14‐I19,I26‐I27.0,I27.2‐I27.9,I28.9‐I29.9,I31.2‐I31.4,I32‐I32.8,I43‐I46.9,I49‐I51,I51.7‐ I59,I62,I62.1,I62.9,I64‐I64.9,I67,I67.4,I67.8‐I69,I69.2,I69.4‐I70.1,I70.9‐I70.92,I79‐I79.8,I90,I92‐I95.1,I95.8‐ I96.9,I98.4,I98.8,I99‐J00.0,J02,J02.8‐J03,J03.8‐J04.31,J06‐J08,J15.9,J17‐J19.6,J22‐J29,J48‐J59,J64‐J64.9,J69‐ J69.9,J71‐J81.9,J83,J85‐J90.9,J93‐J94.9,J96‐K19,K23‐K24,K30,K31.9‐K34,K39,K47‐K49,K53,K54,K58,K63‐K63.4,K63.8 K63.9,K65‐K66.1,K66.9,K69,K71‐K71.2,K71.6,K71.8‐K72.01,K75‐K75.1,K78,K79,K84,K87‐K89,K92.0‐ K92.2,K92.9,K93,K93.1,K93.8,K96‐K99,L06,L07,L09,L15‐L50.9,L52‐L87.9,L90‐L92.9,L94‐L96,L98.5‐L99.8,M04,M10‐ M12.09,M12.2‐M29,M37‐M39,M43.2‐M49,M49.2‐M64,M65.1‐M71,M71.2‐M73,M73.8‐M85.9,M87.2‐ M87.9,M89.1‐M89.49,M90‐M99.9,N09,N13‐N13.9,N17‐N17.9,N19‐N19.9,N24,N32.1,N32.2,N32.8‐N33.8,N35‐ N35.9,N37‐N38,N39.3‐N40.9,N42‐N43.42,N46‐N48.9,N52‐N64.9,N66‐N69,N78,N79,N82‐N82.9,N84,N84.2‐ N86,N88‐N95.9,N97‐N97.9,O08‐O08.9,O17‐O19,O27,O37‐O39,O49‐O59,O78,O79,O93‐O95.9,P06,P16‐P18,P30‐ P34.2,P40‐P49,P62‐P69,P73,P79,P82,P85‐P89,P96.9‐P99.9,Q08‐Q10.3,Q19,Q29,Q36.0‐Q36.9,Q46‐ Q49,Q88,Q89.9,Q94,Q96‐Q96.9,Q98‐Q98.9,Q99.9‐R19.6,R19.8‐R50.1,R50.8,R50.81,R50.84‐R72.9,R74‐R78,R78.6‐ R94.8,R95.0‐T98.3,U04.9,V87‐V87.1,V87.4‐V88.1,V88.4‐ V89.9,V90.00,V99,V99.0,W47,W48,W63,W71,W72,W82,W95‐W97,W98,X07,X41‐X42.9,X44‐X44.9,X55,X56,X59‐ X59.9,Y09‐Y34.9,Y85‐Y87.0,Y87.2,Y89 |
